# Electrocardiographic changes associated with SGLT2 inhibitors and non-SGLT2 inhibitors: A multi-center retrospective study

**DOI:** 10.3389/fcvm.2022.934193

**Published:** 2022-09-06

**Authors:** Victor Chien-Chia Wu, Kai-Pin Chiu, Chun-Li Wang, Chiu-Yi Hsu, Hui-Tzu Tu, Yu-Tung Huang, Chih-Hsiang Chang, Chien-Hao Huang, Chang-Fu Kuo, Shao-Wei Chen, Pao-Hsien Chu, Shang-Hung Chang

**Affiliations:** ^1^Division of Cardiology, Chang Gung Memorial Hospital, Linkou Medical Center, Taoyuan, Taiwan; ^2^Center for Big Data Analytics and Statistics, Chang Gung Memorial Hospital, Linkou Medical Center, Taoyuan, Taiwan; ^3^Department of Nephrology, Kidney Research Center, Chang Gung Memorial Hospital, Linkou Medical Center, Taoyuan, Taiwan; ^4^Division of Hepatology, Department of Gastroenterology and Hepatology, Chang Gung Memorial Hospital, Linkou Medical Center, Taoyuan, Taiwan; ^5^Division of Rheumatology, Allergy and Immunology, Department of Internal Medicine, Chang Gung Memorial Hospital, Linkou Medical Center, Taoyuan, Taiwan; ^6^Division of Rheumatology, Orthopaedics and Dermatology, School of Medicine, University of Nottingham, Nottingham, United Kingdom; ^7^Department of Cardiothoracic and Vascular Surgery, Chang Gung Memorial Hospital, Linkou Medical Center, Taoyuan, Taiwan; ^8^Graduate Institute of Nursing, Chang Gung University of Science and Technology, Taoyuan, Taiwan

**Keywords:** type 2 diabetes mellitus, sodium-glucose co-transporter 2 (SGLT 2) inhibitors, electrocardiogram, QT prolongation, outcome

## Abstract

**Background:**

Sodium-glucose co-transporter 2 (SGLT2) inhibitors has been shown with cardiovascular benefit in type 2 diabetes mellitus (T2DM) patients. However, its osmotic diuresis still concern physicians who may look for possible electrolyte imbalance. We therefore aimed to investigate electrocardiographic (ECG) changes associated with SGLT2 inhibitors.

**Methods:**

Electronic medical records from Chang Gung Research Database between January 1, 2001 and January 31, 2019 were searched for patients with ECG reports and patients on an oral hypoglycemic agent (OHA). We then separate these T2DM patients with EKG into those taking either SGLT2 inhibitors or non-SGLT2 inhibitors. We excluded patients with OHA use <28 days, age <18 years, baseline ECG QTc > 500 ms, and ECG showing atrial fibrillation or atrial flutter. Propensity score matching (PSM) was performed between groups by age, sex, comorbidities, and medications (including QT prolonging medications). Conditional logistic regression and Firth's logistic regression for rare events were employed to compare the difference between SGLT2 and non-SGLT2 inhibitor patients.

**Results:**

After exclusion criteria and PSM, there remained 1,056 patients with ECG on SGLT2 inhibitors and 2,119 patients with ECG on non-SGLT2 inhibitors in the study. There were no differences in PR intervals, QT prolongations by Bazett's or Fridericia's formulas, new onset ST-T changes, new onset CRBBB or CLBBB, and ventricular arrhythmia between the group of patients on SGLT2 inhibitors and the group of patients on non-SGLT2 inhibitors. There were no differences between the two groups in terms of cardiovascular death and sudden cardiac death. In addition, there were no differences between the two groups in terms of electrolytes.

**Conclusions:**

Compared with T2DM patients on non-SGLT2 inhibitors, there were no differences in PR interval, QT interval, ST-T changes, bundle-branch block, or ventricular arrhythmia in the patients on SGLT2 inhibitors. There were no differences in cardiovascular mortality between these two groups. In addition, there were no electrolyte differences between groups. SGLT2 inhibitors appeared to be well-tolerated in terms of cardiovascular safety.

## Introduction

Patients with type 2 diabetes mellitus (T2DM) have increased risks of atherosclerosis and are predisposed to cardiovascular events. Appropriate treatment of diabetes, therefore, does not only hinge on lowering serum glucose level but also drugs that can effectively decrease cardiovascular morbidity and mortality in diabetic patients.

The introduction of sodium-glucose co-transporter-2 (SGLT2) inhibitors as the latest category of antidiabetic agents was evidenced by successful clinical trials of EMPAG-REG, DECLARE-TIMI 58, CANVAS, showing a reduction of major cardiovascular events in patients with T2DM (1–3). The DAPA-Heart Failure (HF) trial published shortly after, showed that the use of dapagliflozin also resulted in decreased hospitalization for HF in patients with reduced ejection fraction, regardless of the presence or absence of diabetes (4).

The versatility of SGLT2 inhibitors, derived their benefits from inhibition of glucose reabsorption from proximal convoluted tubules in kidney and the additional diuresis effect. Within extracellular fluid, the SGLT2 inhibitor causes a 200% interstitial fluid volume reduction compared to plasma volume, while traditional diuretics such as furosemide results in a 78% reduction (5). Therefore, the pharmacologic action of osmotic diuresis by SGLT2 inhibition leads to greater electrolyte-free water clearance from interstitial fluid space than from the circulation, causing relief in congestion, with minimal impact on plasma volume. By reducing interstitial fluid volume greater than plasma volume, the SGLT2 inhibitor provides better control of congestion with minimal impact on arterial filling and perfusion. The natriuresis by SGLT2 inhibition also increases 30% to 60% urinary sodium excretion and 300 mL urine volume per day: equivalent to an approximately 7% reduction in plasma volume after 3 months of treatment (6). SGLT2 inhibitor's osmotic diuresis and natriuresis uniformly reduce body fluid volume to relieve congestion, with minimal impact on blood volume and diminished ECF reduction effect in patients without extracellular fluid retention.

The beneficial effects and increased use of SGLT2 inhibitors are not without concerns, including the rare but serious complication of diabetic ketoacidosis, bone fracture, amputation, and electrolyte imbalance (7). Previously investigators have raised concerns over SGLT2 inhibitors with its sodium inhibition, diuresis, and consequent disturbance of electrolyte balance (8). Through small studies in healthy volunteers, use of dapagliflozin and empagliflozin were reported to not be associated with QT interval prolongations (9, 10). However, whether the same findings may hold true for patients with T2DM using SGLT2 inhibitors are not known. Therefore, in this study we aimed to investigate the QT prolongations in patients using SGLT2 inhibitors.

## Methods

### Data source

In this retrospective cohort study, patient data were obtained from the largest health-care provider in Taiwan, Chang Gung Memorial Hospital System, comprising three tertiary-care medical centers and four major teaching hospitals (11–14). The health care provider has more than 10,000 beds and admits more than 280,000 patients servicing approximately one-tenth of the Taiwanese population each year. The hospital identification number of each patient was encrypted and de-identified to protect their privacy. Therefore, informed consent was waived for this study. The diagnosis and laboratory data could be linked and continuously monitored using consistent data encryption. The institutional review board of Chang Gung Memorial Hospital approved the study protocol (IRB No. 202001017B0).

### Study patients

**Figure 1:**
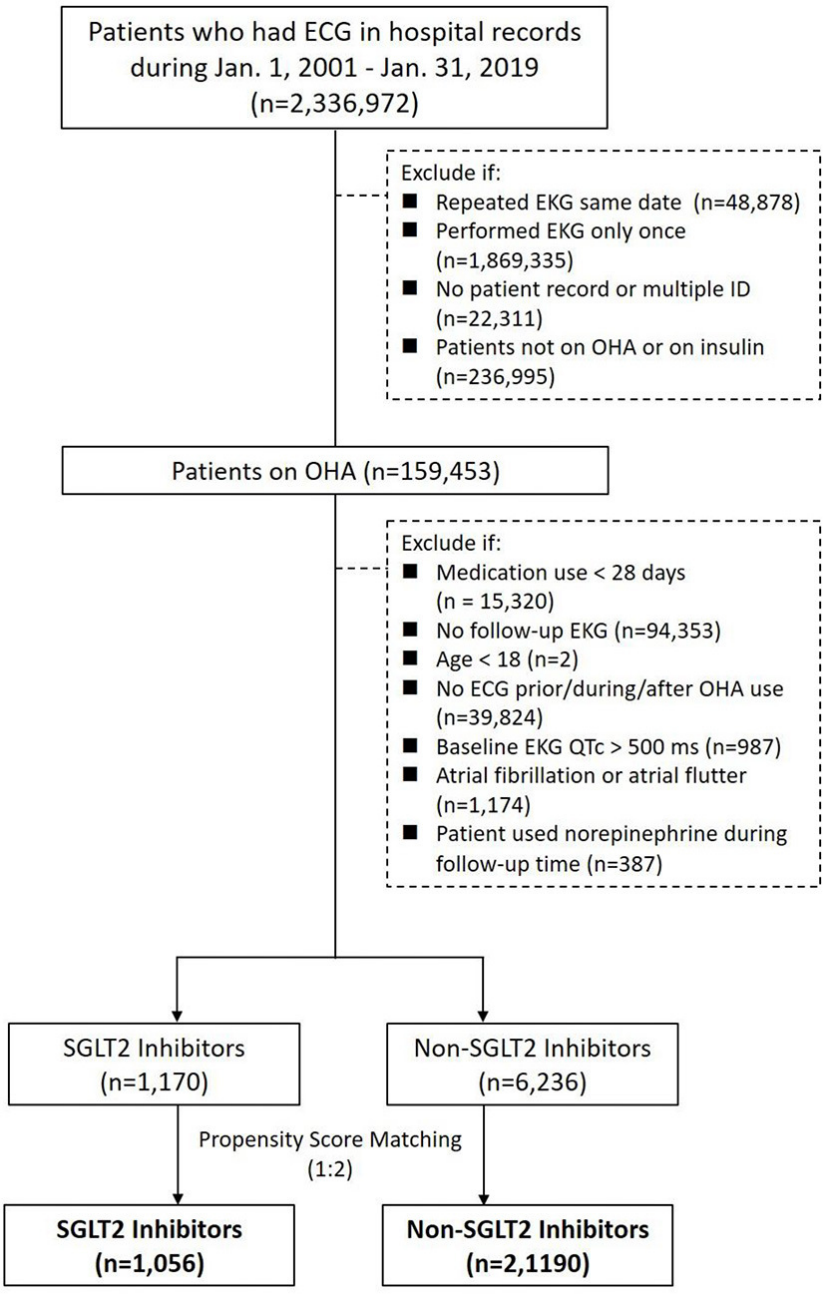
Study design and screening criteria flow chart for the inclusion of patients with T2DM using SGLT2 inhibitors and non-SGLT2 inhibitors with EKG.

**Figure 2:**
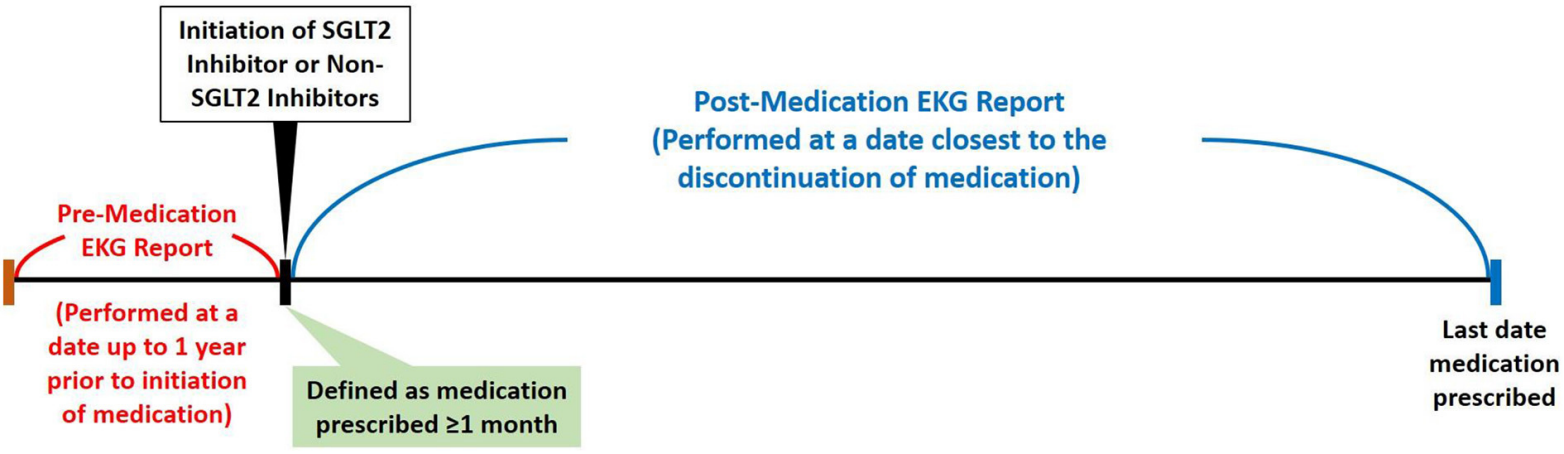
Enrollment criteria for patients with ECG.

By searching electronic medical records from the Chang Gung Research Database (CGRD) between January 1, 2001 and January 31, 2019, we retrieved patient records with electrocardiogram (ECG). We excluded patients with repeated ECG on the same date, had ECG only once, ECG linked to no or multiple hospital identification, and patients not on oral hypoglycemic agent (OHA) or on insulin. We then separate these patients with T2DM and records of ECG into either the group on SGLT2 inhibitors or the group on non-SGLT2 inhibitors. Within each group, we excluded patients that had OHA use <28 days, age <18 years, no ECG prior, during, after OHA use, baseline ECG QTc >500 ms, and ECG showing atrial fibrillation or atrial flutter. Propensity score matching was performed between groups by age, sex, comorbidities, and medications (including QT prolonging medications) (Figure 1). The patients with T2DM enrolled, therefore, had at least 28 days of use of OHA, and we compared the ECGs prior to the use and after the use of OHA such that the first ECG was performed within 1 year prior to the use of OHA and second ECG was performed during the use of OHA (Figure 2).

### Covariate and study outcomes

Disease was detected using International Classification of Diseases, 9th Revision, Clinical Modification (ICD-9-CM) and 10th Revision (ICD-10-CM) codes. Covariates included age, sex, diabetes duration, comorbidity, medications, laboratory data, and follow-up years (Table 1). The comorbidity was defined as having two outpatient diagnoses or one inpatient diagnosis in the previous year. Most diagnostic codes of these comorbidities have been validated in previous national database studies. Usage of medication was retrieved based on claim data in the previous year.

**Table 1:** Clinical characteristics of study population.

**Variable**	**Before propensity score matching**		**After propensity score matching**		
	**SGLT2 inhibitors**	**Non-SGLT2 inhibitors**	**SGLT2 inhibitors**	**Non-SGLT2 inhibitors**	***p*-value**
	**(n = 1,170)**	**(n = 6,236)**	**(n = 1,056)**	**(n = 2,119)**	
	**n, %**	**n, %**	**n, %**	**n, %**	
Age, years	62.54 ± 10.37	66.79 ± 12.24	63.40 ± 10.11	63.69 ± 11.71	0.4589
Male	776 (66.32)	3362 (53.91)	673 (63.73)	1332 (62.86)	0.6316
Diabetes duration, year	7.74 ± 5.3	7.38 ± 5.4	7.98 ± 5.25	6.75 ± 5.25	<0.0001
Comorbidity (n, %)					
Hypertension	875 (74.79)	4619 (74.07)	787 (74.53)	1611 (76.03)	875 (74.79)
Hyperlipidemia	781 (66.75)	3575 (57.33)	704 (66.67)	1413 (66.68)	781 (66.75)
Coronary artery disease	517 (44.19)	1819 (29.17)	423 (40.06)	816 (38.51)	517 (44.19)
Myocardial infarction	391 (33.42)	1247 (20)	310 (29.36)	576 (27.18)	391 (33.42)
Ischemic stroke	50 (4.27)	488 (7.83)	47 (4.45)	90 (4.25)	50 (4.27)
Peripheral artery disease	11 (0.94)	167 (2.68)	11 (1.04)	18 (0.85)	11 (0.94)
Heart Failure	156 (13.33)	702 (11.26)	127 (12.03)	228 (10.76)	156 (13.33)
Atrial fibrillation	26 (2.22)	195 (3.13)	25 (2.37)	48 (2.27)	26 (2.22)
Chronic kidney disease	33 (2.82)	756 (12.12)	31 (2.94)	56 (2.64)	33 (2.82)
Malignancy	124 (10.6)	1213 (19.45)	118 (11.17)	229 (10.81)	124 (10.6)
Medication (n, %)					
QT prolonging agents	428 (36.58)	3650 (58.53)	406 (38.45)	826 (38.98)	0.7712
Alprazolam	116 (9.91)	934 (14.98)	112 (10.61)	227 (10.71)	0.9270
Amiodarone	41 (3.5)	266 (4.27)	40 (3.79)	66 (3.11)	0.3198
Amitriptyline	22 (1.88)	220 (3.53)	21 (1.99)	54 (2.55)	0.3278
Aripiprazole	4 (0.34)	36 (0.58)	4 (0.38)	10 (0.47)	1.0000^a^
Chlorpromazine	5 (0.43)	76 (1.22)	4 (0.38)	15 (0.71)	0.2573
Ciprofloxacin	21 (1.79)	559 (8.96)	21 (1.99)	108 (5.1)	<0.0001
Clozapine	0 (0)	7 (0.11)	0 (0)	3 (0.14)	05553^a^
Dexmedetomidine	3 (0.26)	26 (0.42)	3 (0.28)	5 (0.24)	0.7261^a^
Donepezil	7 (0.6)	92 (1.48)	6 (0.57)	21 (0.99)	0.2215
Dronedarone	5 (0.43)	40 (0.64)	5 (0.47)	7 (0.33)	0.5483^a^
Escitalopram	11 (0.94)	135 (2.16)	11 (1.04)	34 (1.6)	0.2062
Flecainide	13 (1.11)	57 (0.91)	13 (1.23)	13 (0.61)	0.0689
Furosemide	181 (15.47)	1856 (29.76)	164 (15.53)	380 (17.93)	0.0905
Fluconazole	9 (0.77)	141 (2.26)	9 (100)	26 (1.23)	<0.0001^a^
Levetiracetam	9 (0.77)	117 (1.88)	9 (0.85)	11 (0.52)	0.2636
Levofloxacin	2 (0.17)	781 (12.52)	2 (0.19)	130 (6.13)	<0.0001
Lithium	43 (3.68)	9 (0.14)	42 (3.98)	4 (0.19)	<0.0001
Metoclopramide	2 (0.17)	1568 (25.14)	2 (0.19)	315 (14.87)	<0.0001
Mirtazapine	101 (8.63)	84 (1.35)	94 (8.9)	19 (0.9)	<0.0001
Olanzapine	0 (0)	35 (0.56)	0 (0)	6 (0.28)	0.1874^a^
Ondansetron	4 (0.34)	250 (4.01)	4 (0.38)	49 (2.31)	<0.0001
Phenobarbital	26 (2.22)	0 (0)	26 (2.46)	0 (0)	<0.0001
Risperidone	0 (0)	65 (1.04)	0 (0)	11 (0.52)	0.0202^a^
Venlafaxine	1 (0.09)	41 (0.66)	1 (0.09)	10 (0.47)	0.1134^a^
Ziprasidone	2 (0.17)	3 (0.05)	2 (0.19)	1 (0.05)	0.2582^a^
ACEI or ARB	753 (64.36)	3699 (59.32)	667 (63.16)	1351 (63.76)	0.7433
ARNI	35 (2.99)	59 (0.95)	14 (1.33)	37 (1.75)	0.3747
Beta-blockers	725 (61.97)	3579 (57.39)	632 (59.85)	1272 (60.03)	0.9224
Dihydropyridine CCB	71 (6.07)	496 (7.95)	69 (6.53)	126 (5.95)	0.5157
Non-dihydropyridine CCB	73 (6.24)	522 (8.37)	71 (6.72)	130 (6.13)	0.5211
Digoxin	25 (2.14)	128 (2.05)	23 (2.18)	39 (1.84)	0.5172
Ivabradine	23 (1.97)	49 (0.79)	10 (0.95)	23 (1.09)	0.7170
Nitrates	111 (9.49)	707 (11.34)	97 (9.19)	202 (9.53)	0.7523
Diuretics	291 (24.87)	2285 (36.64)	265 (25.09)	517 (24.4)	0.6679
Antiplatelet	673 (57.52)	3126 (50.13)	579 (54.83)	1133 (53.47)	0.4686
Anticoagulant	20 (1.71)	111 (1.78)	19 (1.8)	35 (1.65)	0.7620
Statin	924 (78.97)	3897 (62.49)	818 (77.46)	1656 (78.15)	0.6597
Laboratory (mean ± SD)					
HbA1c, %	8.53 ± 1.65	7.73 ± 1.67	8.51 ± 1.65	7.76 ± 1.66	<0.0001
Hemoglobin	13.57 ± 1.92	12.18 ± 2.21	13.51 ± 1.91	12.95 ± 2.1	<0.0001
Na	139.55 ± 3.31	138.33 ± 5.97	139.57 ± 3.36	138.9 ± 5.42	0.0019
K	4.26 ± 0.58	4.23 ± 0.68	4.26 ± 0.59	4.21 ± 0.5	0.0646
Ca	8.99 ± 0.57	8.88 ± 0.77	9 ± 0.58	8.91 ± 0.8	0.0552
Mg	1.77 ± 0.19	1.77 ± 0.33	1.76 ± 0.19	1.76 ± 0.41	0.9749
Creatinine	0.92 ± 0.36	1.66 ± 2.15	0.92 ± 0.36	1.23 ± 1.41	<0.0001
eGFR	90.71 ± 35.19	75.57 ± 41.41	90.15 ± 35.87	83.76 ± 36.5	<0.0001
AST	31.93 ± 17.8	30.93 ± 22.46	31.93 ± 18.04	30.93 ± 20.49	0.3088
ALT	31.3 ± 21.64	26.58 ± 25.84	30.72 ± 20.04	28.09 ± 23.72	0.0020
Follow-up (years)	1.63 ± 0.77	2.02 ± 0.84	1.64 ± 0.76	2.13 ± 0.77	<0.0001

ACEi, angiotensin converting enzyme inhibitor; ALT, alanine transaminase; ARB, angiotensin receptor blockers; ARNI, angiotensin receptor-neprilysin inhibitor; ASCVD, atherosclerotic cardiovascular disease; AST, aspartate transaminase; BNP, brain natriuretic peptide; CCB, calcium channel blockers; DM, diabetes mellitus; DPP-4i, dipeptidyl peptidase-4 inhibitor; eGFR, estimated glomerular filtration rate; GLP1-RA, glucagon-like peptide 1 receptor agonist; HbA1c, hemoglobin A1c; LVEF, left ventricular ejection fraction; NT-proBNP, N terminal pro B type natriuretic peptide; OHA, other hypoglycemic agent; SGLT2i, sodium glucose co-transporters 2 inhibitor; TZD, thiazolidinedione.

*All statistical tests were 2 tailed, and type I error rate of 0.05 (*p*) is used.

Outcomes of primary interest included PR interval, atrioventricular block (AVB), QT prolongation (Bazett), QT prolongation (Fridericia), new onset ST-T changes, new onset complete right bundle branch block (CRBBB) or complete left bundle branch block (CLBBB), ventricular arrhythmia, and cardiovascular mortality. There are four formulae to correct the QT interval, namely Bazett, Fridericia, Framingham, and Hodges, of which Bazett is the most commonly used and Fridericia is the recommended one in the context of the introduction of new drugs:


$$\begin{array}{l} \text{ Bazett formula: QTc}=\frac{QT}{\sqrt{RR}}\\ \text{Fridericia formula: QTc}=\frac{QT}{\sqrt[{3}]{RR}} \end{array}$$

Each patient was followed until the day of outcome occurrence, date of death or December 31, 2021, whichever came first.

### Statistical analysis

To reduce the potential confounding when comparing outcomes between the study groups (patients on SGLT2 inhibitors vs patients on non-SGLT2 inhibitors), propensity score matching was performed to reduce bias between groups, and the covariates are listed in Table 1. The conditional logistic regression was employed to compare the difference of outcome events between SGLT2 and non-SGLT2 inhibitor group. Moreover, because some outcomes were rare events, the Firth's bias reduction method (sub-type of logistic regression) (15) were used for rare events outcomes. A *P* value < 0.05 was considered to be statistically significant. No adjustment of multiple testing (multiplicity) was made in this study. All statistical analyses were performed using commercial software (SAS 9.4, SAS Institute, Cary, NC).

## Results

### Study population

There were 2,336,972 patient records retrieved with hospital records of ECG between January 1, 2001 and January 31, 2019. After excluding patients with repeated ECG on the same date, had ECG only once, ECG linked to no or multiple hospital identification, and patients not on OHA or on insulin, there were 159,453 patients with ECG on OHA. Since SGLT2 inhibitors became available in Taiwan on May 1, 2016, we identified 21,523 patients with ECG on SGLT inhibitors and 137,930 patients with ECG on non-SGLT2 inhibitors between May 1, 2016 and January 31, 2019. We further excluded patients that had OHA use <28 days, no follow-up ECG, age <18 years, no ECG prior, during, after OHA use, baseline ECG QTc > 500 ms, atrial fibrillation or atrial flutter within each group, and there were 1,170 patients with ECG on SGLT2 inhibitors and 6,236 patients with non-SGLT2 inhibitors. Using 1:2 propensity score matching by age, sex, comorbidities, and medications (including QT prolonging medications), there remained 1,075 patients with ECG on SGLT2 inhibitors and 2,150 patients with ECG on non-SLGT2 inhibitors in the study (Figure 1). Mean diabetes duration of patients on SGLT2 inhibitors was 7.98 ± 5.26 years, and mean diabetes duration of patients on non-SGLT2 inhibitor was 6.75 ± 5.26 years. Mean follow-up of patients on SGLT2 inhibitor was 1.62 ± 0.78 years and mean follow-up of patients on non-SLGT2 inhibitor was 2.11 ± 0.79 years (Table 1).

### Characteristics of ECG of study population

As shown in Table 2, between baseline and follow-up, there were significant differences (but not clinically relevant) of PR interval (1.37 ± 18.38 ms, *p* = 0.00169), QT prolongation by Bazett's formula (2.58 ± 32.17 ms, *p* = 0.0093), and QT prolongation by Fridericia's formula (4.12 ± 30.81 ms, *p* < 0.0001) in patients on SGL2 inhibitors. In addition, between baseline and follow-up, there were significant differences (but not clinically relevant) of PR interval (1.64 ± 18.31 ms, *p* < 0.0001), QT prolongation by Bazett's formula (2.78 ± 31.06 ms, *p* < 0.0001), and QT prolongation by Fridericia's formula (2.10 ± 25.85 ms, *p* = 0.0002) in patients on non-SGL2 inhibitors. However, there were no differences in PR interval, QT prolongation by Bazett's formula, and QT prolongation by Fridericia's formula between groups of patients on SGLT2 inhibitors and patients on non-SGLT2 inhibitors.

**Table 2:** ECG characteristics of study population.

**EKG characteristics**	**SGLT2 Inhibitors**	***p*-value^a^**	**Non-SGLT2 Inhibitors**	***p*-value^a^**	***p*-value^b^**
	**Mean ±SD**		**Mean ±SD**		
PR Interval		0.0169		<0.0001	0.6954
Baseline PR interval, ms	170.28 ± 28.76		167.23 ± 28.05		
Follow-up PR interval, ms	171.39 ± 29.57		168.81 ± 29.66		
PR interval difference, ms	1.37 ± 18.38	–	1.64 ± 18.31	–	
QT prolongation, Bazett's formula		0.0093		<0.0001	0.8680
Baseline QTc, ms	435.03 ± 33.10		434.28 ± 29.99		
Follow-up QTc, ms	437.61 ± 27.75		437.05 ± 29.34		
QTc difference, ms	2.58 ± 32.17	–	2.78 ± 31.06	–	
QT prolongation, Fridericia's formula		<0.0001		<0.0002	0.0687
Baseline QTc, ms	417.51 ± 30.57		418.25 ± 24.91		
Follow-up QTc, ms	421.57 ± 25.18		420.26 ± 26.55		
QTc difference, ms	4.12 ± 30.81	–	2.10 ± 25.84	–	

*Difference: between follow-up and baseline.

a Paired *t* test.

b Independent *t* test.

### ECG outcomes of study population

#### PR interval

As shown in Table 3, in terms of PR interval, there was no difference between group of patients on SGLT2 inhibitors and group of patients on non-SGLT2 inhibitors in terms of first degree AVB. In addition, there was no difference between the two groups in terms of type II second degree AVB or complete AVB.

**Table 3:** ECG outcomes of study population.

**Outcome**	**SGLT2 Inhibitors**	**Non-SGLT2 Inhibitors**	**OR (95% CI)**	***p*-value**
	**n, %**	**n, %**		
PR Interval				
First degree AVB	96 (9.09)	179 (8.45)	1.086 (0.838 to 1.408)	0.5323
Type II second degree AVB or complete AVB	1 (0.09)	4 (0.19)	0.668 (0.105 to 4.249)	0.6692
QT prolongation, Bazett's formula				
QTc > 440 ms in men	245 (23.20)	489 (23.08)	1.008 (0.846 to 1.200)	0.9314
QTc > 460 ms in women	95 (9.00)	169 (7.98)	1.143 (0.879 to 1.486)	0.3184
QTc > 500 ms	16 (1.52)	55 (2.60)	0.590 (0.339 to 1.027)	0.0622
QTc <350 ms	2 (0.19)	0 (0)	10.049 (0.482 to 209.663)	0.1366
Increase in QTc >60	19 (1.80)	53 (2.50)	0.726 (0.430 to 1.226)	0.2313
QT prolongation, Fridericia's formula				
QTc > 440 ms in men	112 (10.61)	220 (10.38)	1.026 (0.807 to 1.305)	0.8329
QTc > 460 ms in women	32 (3.03)	72 (3.40)	0.896 (0.588 to 1.364)	0.6085
QTc > 500 ms	6 (0.57)	16 (0.76)	0.789 (0.317 to 1.962)	0.6099
QTc <350 ms	2 (0.19)	2 (0.09)	2.008 (0.347 to 11.616)	0.4363
Increase in QTc >60	21 (1.99)	43 (2.03)	0.991 (0.588 to 1.671)	0.9733
New onset ST-T changes	0 (0)	3 (0.14)	0.286 (0.015 to 5.553)	0.4083
New onset CRBBB or CLBBB	5 (0.47)	22 (1.04)	0.488 (0.191 to 1.243)	0.1324
Ventricular Arrhythmia	13 (1.23)	14 (0.66)	1.879 (0.892 to 3.958)	0.0973
Ventricular Tachycardia	13 (1.23)	14 (0.66)	1.879 (0.892 to 3.958)	0.0973
Ventricular Fibrillation	0 (0)	0 (0)	–	–
Torsades de Pointes	0 (0)	0 (0)	–	–

AVB, atrioventricular block; CRBBB, complete right bundle branch block; CLBBB, complete left bundle branch block; OR, odds ratio; CI, confidence interval.

#### QT interval

In terms of QT prolongation by Bazett's formula, including men with QTc > 440 ms, women with QTc > 460 ms, QTc > 500 ms, QTc <350 ms, or increase in QTc > 60 ms, there were no differences between the two groups. In terms of QT prolongation by Fridericia's formula, including men with QTc > 440 ms, women with QTc > 460 ms, QTc > 500 ms, QTc <350 ms, or increase in QTc > 60 ms, there were no differences between the two groups.

#### ST-T changes

In terms of new onset ST-T changes, there was no difference between the two groups.

#### Bundle-branch block

In terms of new onset complete right bundle branch block (CRBBB) or complete left bundle branch block (CLBBB), there was no difference between the two groups.

#### Ventricular arrhythmia

In terms of ventricular arrhythmia, including ventricular tachycardia, ventricular fibrillation, or Torsades de Pointes, there were no differences between the two groups.

### Mortality outcome of study population

In terms of cardiovascular mortality, including cardiovascular death and sudden cardiac death, there were also no differences between the two groups (Table 4).

**Table 4:** Mortality outcomes of study population.

**Outcome**	**SGLT2 Inhibitors n, %**	**Non-SGLT2 Inhibitors**	**OR (95% CI)**	***p*-value**
		**n, %**		
Cardiovascular mortality	4 (0.37)	13 (0.58)	0.665 (0.228 to 1.938)	0.4547
Cardiovascular Death	1 (0.09)	3 (0.13)	0.857 (0.126 to 5.813)	0.8741
Sudden cardiac death	3 (0.28)	8 (0.36)	0.823 (0.236 to 2.864)	0.759

OR, odds ratio; CI, confidence interval.

### Electrolyte outcomes of study population

As shown in Table 5, between baseline and follow-up electrolyte levels, the group of patients on SGLT2 inhibitors had a significant increase (but not clinically relevant) in sodium (0.66 ± 4.06 mEq/L, *p* = 0.0009), but no difference in potassium (0.04 ± 1.17 mEq/L, *p* = 0.3771), calcium (0.002 ± 0.98 mg/dL, *p* = 0.9836), and magnesium (0.06 ± 0.25 mEq/L, *p* = 0.3306). On the other hand, between baseline and follow-up electrolyte levels, the group of patients on non-SGLT2 inhibitors had no difference in sodium (0.32 ± 7.77 mEq/L, *p* = 0.2083), but a significant increase (but not clinically relevant) in potassium (0.05 ± 0.67 mEq/L, *p* = 0.0096), calcium (0.12 ± 1.08 mg/dL, *p* = 0.0404), and magnesium (0.08 ± 0.24, *p* = 0.0456). There was overall no difference of electrolyte levels between groups.

**Table 5:** Electrolyte outcomes of study population.

**Laboratory**	**SGLT2 Inhibitors**	***p*-value^a^**	**Non-SGLT2 Inhibitors**	***p*-value^a^**	***p*-value^b^**
	**Mean ±SD**		**Mean ±SD**		
Na		0.0009		0.2083	0.3017
Baseline	139.56 ± 3.36		138.91 ± 5.4		
Follow-up	140.26 ± 3.47		139.15 ± 5.48		
Na difference	0.66 ± 4.06	–	0.32 ± 7.77	–	
K		0.3771		0.0096	0.8452
Baseline	4.26 ± 0.59		4.21 ± 0.5		
Follow-up	4.3 ± 0.94		4.26 ± 0.54		
K difference	0.04 ± 1.17	–	0.05 ± 0.67	–	
Ca		0.9836		0.0404	0.3456
Baseline	9.01 ± 0.58		8.91 ± 0.8		
Follow-up	9.04 ± 0.72		8.99 ± 0.75		
Ca difference	0.002 ± 0.98		0.12 ± 1.08		
Mg		0.3306		0.0456	0.7498
Baseline	1.77 ± 0.19		1.76 ± 0.4		
Follow-up	1.82 ± 0.29		1.79 ± 0.32		
Mg difference	0.06 ± 0.25	–	0.08 ± 0.24	–	

*Difference: between follow-up and baseline.

a Paired *t* test.

b Independent *t* test.

## Discussion

This is the first study to compare the changes of ECG of patients on SGLT2 inhibitors and patients on non-SGLT2 inhibitors in a large T2DM patient cohort. There were no differences in PR interval, AV conduction block, QT prolongation (by Bazett's or Fridericia's formulas), new onset ST-T changes, new onset CRBBB or CLBBB, ventricular arrhythmia, and cardiovascular mortality between the groups.

Diabetes is a chronic metabolic disease and causes a complex myocardial dysfunction, referred to as diabetic cardiomyopathy, which even in the absence of other cardiac risk factors results in abnormal diastolic and systolic function (16). Altered electrical function is a major feature of the diabetic myocardium alongside mechanical abnormalities (16). Diabetic patients often exhibit cardiac electrical remodeling, primarily a prolonged ventricular repolarization visible in the electrocardiogram as a lengthening of the QT interval duration secondary to alterations on the expression and activity of several cardiac ion channels and their associated regulatory proteins (16). The changes in sodium, calcium, and potassium currents can together lead to a delay in repolarization that increase the risk of developing life-threatening ventricular tachycardia, Torsades de Pointes, and ventricular fibrillation (16). Since QT prolongation is a qualitative marker of proarrhythmic risk, a thorough QT/QTc (TQT) analysis evaluating QT interval prolongation is often performed to assess potential proarrhythmic effects during new drug administration. In light of diabetic patients often have a higher risk of cardiovascular events, cardiovascular safety of the new antidiabetic drugs must be carefully assessed in these T2DM patients.

In a randomized, placebo-controlled, double-blind, four-period crossover study at a single-center inpatient clinical pharmacology unit, 50 healthy men were to receive doses of dapagliflozin 150 mg, dapagliflozin 20 mg, moxifloxacin 400 mg, and placebo (9). Digital 12-lead electrocardiograms were recorded and QT intervals were corrected for heart rate using a study-specific correction factor (QTcX) and Fridericia's formula (9). For dapagliflozin, the upper bound of the one-sided 95% confidence interval (CI) for time-matched, placebo-subtracted, baseline adjusted QTc intervals (ΔΔQTc) was <10 ms of dapagliflozin had little effect on heart rate (9). The results showing that dapagliflozin, at supratherapeutic doses, does not have a clinically significant effect on the QT interval in healthy subjects (9).

In another randomized, placebo-controlled, single-dose, double-blind, five-period crossover study, 30 volunteers were randomized to receive single empagliflozin doses of 25 mg (therapeutic) and 200 mg (supratherapeutic), matching placebo, and open-label moxifloxacin 400 mg (positive control) (10). Triplicate 12-lead ECGs of 10 second duration were recorded at baseline and during the first 24 h after dosing (10). The findings showed that single doses of empagliflozin 25 mg and 200 mg were not associated with QTc prolongation and were well tolerated (10).

In a recent study, the risk of new-onset arrhythmias (NOA) and all-cause mortality with the use of SGLT2 inhibitors were investigated using the national health insurance database (17). Patients with T2DM taking SGLT2 inhibitors were compared to patients with T2DM without taking SGLT2 inhibitors using 1:1 propensity score matching (17). The results showed that compared to 79,150 T2DM patients not taking SGLT2 inhibitors, 79,150 T2DM patients on SGLT2 inhibitors were associated with a lower risk of all-cause mortality (adjusted hazard ratio [aHR] 0.547; 95% confidence interval [CI] 0.482–0.621; *P* = 0.0001) and NOA (aHR 0.830; 95% CI 0.751–0.916; *P* = 0.0002) (17).

A group of researchers recently investigated the effects of SGLT2 inhibitors as an add-on therapy to metformin on electrocardiographic indices of ventricular repolarization in 141 consecutive patients (18). After the six-month follow-up, there was a significant decrease in the QT interval in patients who were using SGLT2 inhibitors as an add-on therapy to metformin compared to other glucose-lowering agents (SGLT2 inhibitors: 373.4 ± 9.9 ms vs. dipeptidyl peptidase-4 inhibitors: 385.4 ± 12.5 ms, sulfonylureas: 382.9 ± 11.2 ms; *p* < 0.001 respectively) (18). The authors concluded that using SGLT2 inhibitors as an add-on therapy to metformin favorably alters ventricular repolarization indices in patients with T2DM (18). In another study the researchers studied the class effects of SGLT2 inhibitors in mouse cardiomyocytes and found that SGLT2 inhibitors directly inhibit cardiac Na^+^/H^+^ exchanger flux and reduce cardiac cytosolic [Na^+^], possibly by binding with the Na^+^-binding site of Na^+^/H^+^ exchanger (19). SGLT2 inhibitors also affect the healthy heart by inducing vasodilation (19). The cardiac cytosolic [Na^+^]-lowering class effect of SGLT2i is a potential approach to combat elevated cardiac cytosolic [Na^+^] known to occur in heart failure and diabetes (19).

In a recent review of mineral and electrolyte disorders with SGLT2 inhibitor therapy, there were the postulated effects of SGLT2 inhibitors on serum electrolytes (sodium, potassium, and magnesium) that inhibition of SGLT2 receptors promotes glycosuria, natriuresis, and osmotic diuresis, which in turn causes an elevation of aldosterone activity with increased kaliuresis and magnesuria (20). These effects are counterbalanced by an improvement in glycemic control with an elevation of serum glucagon and reduction of insulin, which favors redistribution of potassium and magnesium in cells from the intracellular space with a net effect of a potential low increase of serum potassium and magnesium concentrations (20).

In this study, our enrolled patients were propensity score matched between patients on SGLT2 inhibitor and patients on non-SGLT2 inhibitors, including age, sex, comorbidities, medications, especially those QT prolonging agents. For ECG characteristics, between baseline and follow-up, there were minute increases in PR interval, QT prolongation by Bazett's formula, and QT prolongation by Fridericia's formula in both SGLT2 inhibitor group and non-SGLT2 inhibitors group that may be related to diabetic cardiomyopathy, albeit not clinically relevant. Between groups of patients, there were also no differences in PR interval and QT prolongation by Bazett's formula or Fridericia's formula.

In brief, for ECG outcomes, there was no difference between group of patients on SGLT2 inhibitors and group of patients on non-SGLT2 inhibitors in terms of PR intervals, QT prolongations, new onset ST-T changes, new onset CRBBB or CLBBB, or ventricular arrhythmia. For mortality outcomes, there were no differences between the two groups in terms of cardiovascular death and sudden cardiac death. And for electrolyte outcomes, there were also no difference between the two groups in sodium, potassium, calcium, and magnesium levels at follow-up lab tests, which may be associated with no increased morality associated with SGLT2 inhibitors compared to non-SGLT2 inhibitors. These results are in line with previous literature, that SGLT2 inhibitors are relatively safe, do not cause sodium, potassium, calcium, nor magnesium level imbalance, and may have mortality benefits (19, 21–23). To summarize, our study showed stable ECG changes in these patients and offered clinical evidence to the electrocardiographic and cardiovascular safety of SGLT2 inhibitors for treatment of patients with T2DM.

## Limitations

There are several limitations in epidemiologic data from CGRD. First, using ICD-9-CM and ICD-10-CM codes for patient screening and enrollment may have missed some cases for which conditions were coded incorrectly. A second limitation occurs when ECG measurements are performed automatically by the ECG machine as there is no manual validation of the results from automatic measurements. Third, due to a limited number of patients where SGLT2i was prescribed as monotherapy, there may be not enough patient data to decrease the range of days the ECG was acquired to the use of OHA. Due to small number of patients on SGLT2 inhibitors, we did not discern each SGLT2 inhibitor for the individual outcomes against non-SGLT2 inhibitors. Last, since our study consisted of nearly homogenous racial background, application of the results to other populations requires further studies.

## Conclusion

Compared with T2DM patients on non-SGLT2 inhibitors, there was no difference in PR interval, QT interval, ST-T changes, bundle-branch block, or ventricular arrhythmia in the patients on SGLT2 inhibitors. There was no difference in cardiovascular mortality between these two groups. In addition, there were no electrolyte difference between groups. SGLT2 inhibitors appeared to be well-tolerated in terms of cardiovascular safety.

## Data availability statement

The raw data supporting the conclusions of this article will be made available by the authors, without undue reservation.

## Ethics statement

The studies involving human participants were reviewed and approved by the Institutional Review Board of Chang Gung Memorial Hospital approved the study protocol (IRB No. 202001017B0). The Ethics Committee waived the requirement of written informed consent for participation.

## Author contributions

Study conception and design: VC-CW, K-PC, C-LW, and S-HC. Acquisition of data: C-YH, H-TT, and Y-TH. Analysis and interpretation of data: C-HC, C-HH, C-FK, and S-WC. Drafting of manuscript: VC-CW and K-PC. Critical revision: C-LW, P-HC, and S-HC.

## Funding

This work was supported by the grant from Chang Gung Memorial Hospital (CMRPG3I0093 and CLRPG3D0049).

## Conflict of interest

The authors declare that the research was conducted in the absence of any commercial or financial relationships that could be construed as a potential conflict of interest.

## Publisher's note

All claims expressed in this article are solely those of the authors and do not necessarily represent those of their affiliated organizations, or those of the publisher, the editors and the reviewers. Any product that may be evaluated in this article, or claim that may be made by its manufacturer, is not guaranteed or endorsed by the publisher.

## Abbreviations

AVB: atrioventricular block
CLBBB: complete left bundle branch block
CRBBB: complete right bundle branch block
ECG: electrocardiogram
HF: heart failure
OHA: oral hypoglycemic agent
SGLT2: sodium-glucose co-transporter-2
T2DM: type 2 diabetes mellitus.
